# The Supply of Lime Phosphates to the Mother during Periods of Gestation and Lactation

**Published:** 1872-06

**Authors:** A. O. Rawls


### The Supply of Lime Phosphates to the Mother During Periods of Gestation and Lactation.

BY A. O. RAWLS.

There is probably no subject pertaining to either dental or general therapeutics of more vital importance or fraught with greater essential interest to both medical and dental practitioner, and at the same time less thoroughly appreciated by them, than the subject chosen for our first consideration before this society. While in our limited knowledge of the subject we feel somewhat embarrassed in attempting to advance any point of special or common interest, and entirely inadequate to the task of adding any thing new, yet we may be permited to enjoy, even with satisfaction, the performance of a duty alike incumbent on each and every one who are in any wise connected with the healing art, and contribute cheerfully our mite, though small, toward bringing the subject before this society in a manner that will elicit from each one an opinion, thereby preparing the way for a more consumate elucidation of facts and theories, in all their bearings, having reference whatever to the issue before us. It is a well-known, indeed, a most deplorable fact, that thousands upon thousands of our race, old and young, of either sex, are enervating their systems, destroying their health, and in a manner, sapping the very fountain-head of their existence by perversely ignoring some of the simple and yet most useful rules of dietetics. Although from observation and statistics we have reason to believe that among the masses of more intelligent society, there is being wrought a material change for good, and that our public lecturers, books, periodicals, medical and dental instructors, laboring in this field, are exerting considerable influence for the welfare of those that heed the warning, still there remains ample room for future revolution in this direction in order that all may come to a knowledge of the truth and reap the benefits accruing from the performance of a well known duty. Our subject, however, refers particularly to mothers, and to them more particularly who are laboring under the exhaustive influence consequent upon, and following the periods of gestation and lactation. Now there are few if any authors that advocate the use of any special diet as indicated for the mother during these periods of her existence, and possibly leave no assurance, or established fact proven by experiment, that it were possible for the powers of digestion, absorption, and assimilation, to be sufficiently active in order that the supply of nutriment might be commensurate to the entire demand of the system for the same. However this may be, we have general principles governing this as well as other subjects relating to each peculiar phase of life, and so accordingly must resort to general principles before we can hope to meet, with success, demands of a special character.

When we would speak of the supply of lime phosphates to the mother during the periods referred to, it of necessity leads us to a consideration of the subject in a much broader sense than simply supplying this article of diet to her for consumption, it leads us to a consideration of its proper admixture with other articles of diet, and to consider the subjects of mastication, absorption, and assimilation, as they refer to articles of diet in general, but with more special reference to the employment of lime phosphates; it also leads us to an observation of the circumstances and condition of each individual patient, such as their habits, temperament, quantity and quality of food usually taken, and that best suited to their respective conditions, during these periods referred to. An adequate and proper supply of material for the replacement of broken down tissue, in any part of the body, depends greatly upon the habits, diet, quality and quantity of food, mastication, insalivation, and on the comparative health of all functions concerned in this reparation. Now as it is our province to deal more especially with those organs whose greatest honor is to consumate the first metamorphosis of food destined to become a part and parcel of our existence, we will refer briefly to the process of mastication, upon the good and perfect performance of which much depends in the way of assisting other functions to perform their respective duties with promptness and with regularity, thus enabling all parts to act in harmony and strength together. The thorough and complete mastication of fooa has a direct two-fold purpose toward aiding digestion and assisting the powers of absorption and assimilation. First, to bring into action the dental organism to such a degree as will best promote the health and strength of their special functions, just as the proper exercise and judicious use of any portion of the body would add to its health and strength, and so accordingly be an important adjunct to the system in general, by lessening the number of pathological conditions usually accompanying the periods of gestation and lactation, that would render nutrition imperfect; secondly, perfect mastication not only allows sufficient time for a necessary insalivation of food (especially requisite for all amylacious articles), but it produces such a condition of comminution in the mass of food as tends to greatly increase the surface of the same, thereby presenting it to the action of gastric and other solvent juices in a shape and compatibility that will render the process of digestion in that part of the alimentary canal more expeditious and complete, thus preparing it the better for each successive change, until absorbed and carried into the general circulation to be assimilated as required at different points throughout the entire system. There is one other point in connection with this part of our subject which I desire to call your attention to, viz: that the conditions of the dental organism has very much to do with the selection of a diet, and as a proof of this we will find that it is not an uncommon observation by even the most causual observers that persons whose teeth are badly decayed, poorly antagonized, or rendered sensitive from any of the various causes, usually confine themselves to a vegetable, and, as they suppose, more suitable diet for their imperfect powers of mastication. The effects of this course of dietetics for ill results are obvious to those understanding and appreciating the necessity for articles of food containing greater varieties of elementary atoms constituting tissues of our body. Thus far we have been able to observe the importance of good teeth and perfect mastication as among the first requirements favoring digestion and assimilation of food.

Now by what means can we best supply lime phosphates to meet this extraordinary metamorphosis of tooth and bone structure? Most assuredly just as we would supply any other article of complementary diet. It is a well founded fact that we can not live any great length of time and retain our usual health and vigor on any single diet, the system, after a time revolting, refuses to allow its absorption, much less assimilation, in quantities that under more favorable circumstances would prove insufficient for the demanded supply. In this then we have exhibited the necessity for a changing and various diet. If the tissues of our body are composed of certain elementary principles, then we can not do otherwise, if we desire health and strength, than meet the waste of such tissues with elements of their natural structure; and to accomplish this object successfully there must exist a just and proper proportion of these atoms varying to suit each individual case. Although an article or articles of food may possess all elementary atoms that are contained within this or that structure of the system, such as hydrogen, nitrogen, carbon, sulphur, salts, water, and so on, yet it is by no means conclusive that the waste will be supplied, for there may be an undue proportion, thereby limiting its assimilation to certain and inadequate amounts, or as regards quantity there may be too much, in which case the system is over-taxed in order that it may rid itself of the surplus; and while evil results may follow an over loaded stomach, yet in all probability the true mode of dieting as regards quantity is to favor a little surplus rather than deficiency in amount. As a rule mothers during the periods of gestation and lactation are so broken in systemic vigor that it seems impossible at times for food enough to be absorbed and assimulated, so that they may be enabled to retain even an ordinary healthy condition of structural parts. When this is the case their duty consists in making use of all available means for the restoration of their usual vigor, such as moderate out-door exercise, walking, riding, anything in this direction, so they are exposed to sunlight, and are enabled to enjoy the benefit of fresh air, and the exercise is not continued to exhaustion, or of a character too laborious. And again since the mind exerts an extensive influence over the process of digestion and assimulation it should be as free possible from troublesome thought; they should avoid severe ex ercise, either mental or physical, right after meals, and if the digestive apparatus is weakened from any cause, their meals should be oftner than usual, the quantity taken lessened, and taken at regular intervals. In short, the strict observance of all hygenic rules coming to their knowledge is of very necessary and vital importance. When the system is thus indeed fortified by an observation of the rules increasing health and strength, there need be nɔ fear that rapid metamorphosis of tissue will materially interfere with its structural identity, if articles of food containing all necessary constituents are only supplied in proper quantities and admixture with each other. In conclusion I will add that arresters of metamorphosis in the tissues, such as sugar, and all acholic drinks, tea, and coffee, might exert a favorable influence at times toward staying the ravages made upon the mother system during these periods.

